# Eosinophils and chronic obstructive pulmonary diseases (COPD) in hospitalized COVID-19 patients

**DOI:** 10.1186/s12879-024-09373-2

**Published:** 2024-06-03

**Authors:** Mitra Samareh Fekri, Zohreh Najminejad, Fatemeh Karami Robati, Behnam Dalfardi, Mahdiyeh Lashkarizadeh, Mohammad Javad Najafzadeh

**Affiliations:** 1 Cardiovascular Research Center, Institute of Basic and Clinical Physiology Sciences, Kerman University of Medical Sciences, Kerman, Iran; 2 Clinical Research Development Unit, Afzalipour Hospital‚ Kerman University of Medical Sciences, Kerman, Iran; 3 Advanced Thoracic Research Center, Tehran University of Medical Sciences, Tehran, Iran; 4 Department of Pathology and Stem Cell Research Center, School of Medicine, Kerman University of Medical Sciences, Kerman, Iran; 5 Student Research Committee, Kerman University of Medical Sciences, Kerman, Iran

**Keywords:** COPD, COVID-19, Eosinophils, Mortality

## Abstract

**Background:**

The emergence of coronavirus disease 2019 (COVID-19) as a global health emergency necessitates continued investigation of the disease progression. This study investigated the relationship between eosinophilia and the severity of COVID-19 in chronic obstructive pulmonary disease (COPD) patients.

**Methods:**

This cross-sectional study was conducted on 73 COPD patients infected by COVID-19 in Afzalipour Hospital, Iran. Peripheral blood samples were collected for hematological parameter testing, including eosinophil percentage, using Giemsa staining. Eosinophilia was defined as≥ 2% and non-eosinophilia as< 2%. The severity of pulmonary involvement was determined based on chest CT severity score (CT-SS) (based on the degree of involvement of the lung lobes, 0%: 0 points, 1–25%: 1 point, 26–50%: 2 points, 51–75%: 3 points, and 76–100%: 4 points). The CT-SS was the sum of the scores of the five lobes (range 0–20).

**Results:**

The average age of patients was 67.90±13.71 years, and most were male (54.8%). Non-eosinophilic COPD patients were associated with more severe COVID-19 (*P=* 0.01) and lower oxygen saturation (*P=* 0.001). In addition, the study revealed a significant difference in the chest CT severity score (CT-SS) between non-eosinophilic (9.76±0.7) and eosinophilic COPD patients (6.26±0.63) (*P*< 0.001). Although non-eosinophilic COPD patients had a higher mortality rate, this difference was not statistically significant (*P=* 0.16).

**Conclusions:**

Our study demonstrated that reduced peripheral blood eosinophil levels in COPD patients with COVID-19 correlate with unfavorable outcomes. Understanding this association can help us identify high-risk COPD patients and take appropriate management strategies to improve their prognosis.

## Background

COVID-19 predominantly affects the respiratory system, with highly variable clinical manifestations ranging from minimal flu-like symptoms to considerable hypoxia and acute respiratory distress syndrome [1]. Systemic manifestations are common in severe COVID-19, and endothelial cell damage causes cardiac, renal, and neurological complications [2]. All age groups are at risk of infection, although elderly persons (> 65 years) are more at risk. Coronary artery disease, diabetes, and hypertension have been identified as risk factors for mortality [3].

Severe acute respiratory syndrome coronavirus 2 (SARS-CoV-2) may cause exacerbation in chronic obstructive pulmonary disease (COPD) patients. In addition, the incidence of hospitalization and severity of illness in patients with pre-existing respiratory diseases, such as COPD, is much higher in patients with COVID-19 than with seasonal influenza [4].

Considering that the global prevalence of COPD was 10.3% in 2019 [5], it is critical to study the effects of COVID-19 on this group of patients. People with COPD are potentially more susceptible to severe consequences of COVID-19 because viral infections that affect the upper or lower airways are some of the leading causes of hospitalization and exacerbations in these patients [6]. The results of the studies by Guan et al. and Docherty et al. showed that COPD is associated with severe COVID-19 outcomes [7, 8]. Bafadhel et al. found that high blood eosinophil levels in COPD patients are associated with an increased risk of exacerbations [9].

Eosinophils play a significant role in the pathogenesis of various respiratory diseases, including COPD and asthma. Eosinophils comprise a small percentage (1–3%) of all circulating leukocytes [10]. In recent years, eosinophil levels in peripheral blood have become one of the emerging biomarkers in patients with COPD because there is evidence that eosinophils participate in the pathophysiological mechanisms of the disease, and their measurement is possible in most laboratories [11]. However, the effect of eosinophils in stable COPD and COPD exacerbations may differ [12].

In a cohort study, Lucas et al. observed an increase in eosinophil levels among patients with severe COVID-19 [13]. In contrast, many recent studies have reported a significant decrease in eosinophil levels in COVID-19 patients [14, 15]. COVID-19 may deplete CD8 T-cells, which normally produce IL-5 [16]. IL-5 contributes to the proliferation and activation of eosinophils [17]. IL-5 and IL-13 are produced by T helper 2 (T_H_2) cells. The role of T_H_2 cells in severe COVID-19 remains unclear [18].

This study evaluated the association between eosinophilia levels in peripheral blood and the severity of COVID-19 in COPD patients.

## Methods

### Study design and participants

**Fig. 1:**
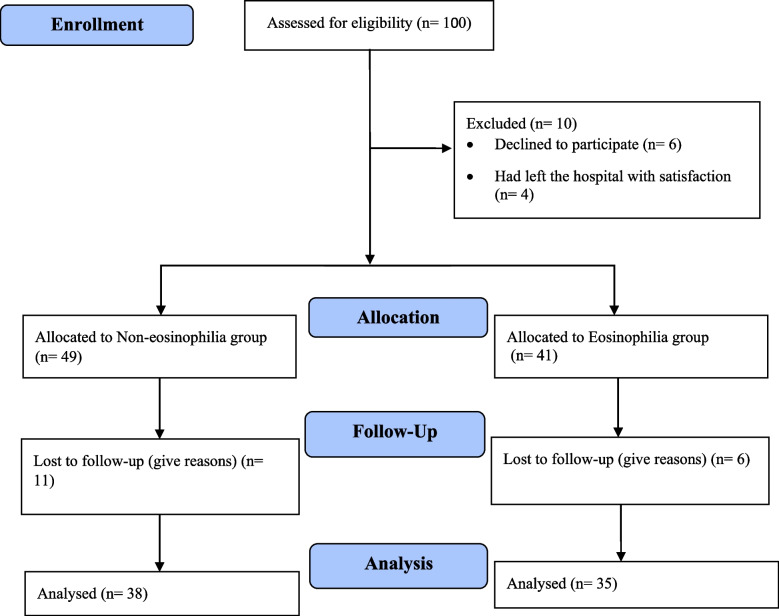
CONSORT Flow Diagram

This cross-sectional study was conducted on COPD inpatients infected by SARS-CoV-2 at Afzalipour Hospital, Kerman, Iran, from January to July 2022. According to reference [14], in people with COVID-19, the frequency of eosinopenia was 53%, with an error of d=0.3, *p*=0.05 and α=0.05, the sample size was equal to 50 people using the sample size formula for a population. In order to improve the results, increase the statistical power of the test and the possibility of dropping samples, 100 people were examined. Then six patients declined to participate in study and four patients had left the hospital with satisfaction. After that, 49 and 41 people were allocated to non-eosinophilia and eosinophilia group. 11 people in non-eosinophilia group and 6 people in eosinophilia group were lost to follow-up. Finally, 73 people remained in the study (38 in non-eosinophilia and 35 in eosinophilia group) (Fig. 1).

The patients were selected by random sampling. The inclusion criteria were age 40 years and older, positive throat and nose reverse transcriptase polymerase chain reaction (RT-PCR) test for COVID-19, history of COPD, and informed consent to enter the study. Also patients with clinical signs of pneumonia (i.e., fever, cough, and dyspnea) and SPO2≥ 90% on room air were classified as non-severe cases and patients with clinical signs of pneumonia (i.e., fever, cough, and dyspnea) and at least one of the following signs were classified as severe cases: respiratory rate> 30 breaths/minute, severe respiratory distress, SpO2 < 90% on room air, shock, or other organ failures included in study. The exclusion criteria were known immunodeficiency (patients who used immunosuppressive drugs before the diagnosis of COVID-19), previous diagnosis or clinical symptoms consistent with asthma, cancer, oral corticosteroid use before hospitalization, and lack of cooperation or consent to participate in the study.

### Data collection

Immediately after positive PCR test, a 7-cc peripheral blood sample was taken from each hospitalized COPD patient infected by COVID-19 to test hematological parameters, including erythrocyte sedimentation rate (ESR), white blood cells (WBC), lactate dehydrogenase (LDH), D-dimer, hemoglobin (HB), hematocrit (HCT), mean corpuscular volume (MCV), platelets (PLT), neutrophils (NEUT%) and lymphocytes (LYMPH%). Peripheral blood slides were stained using Giemsa staining to determine the eosinophil percentage. Oxygen saturation on admission and discharge days (SPO_2_1 and SPO_2_2, respectively) was measured by pulse oximetry when the patient did not receive oxygen therapy.

The patient’s demographic information, including age, gender, history of smoking and inhaling opium, past medical history, oxygen therapy methods, CT scan information, and treatment outcomes, was extracted from patients’ records.

Chest CT was performed using a Philips Diamond Select Brilliance CT scanner (made in the USA). The radiologists reported the CT scans, then an expert pulmonologist who was blinded to the patients’ laboratory results, read and reported all the CT scans based on the scoring of reference [19].

The severity of pulmonary involvement was determined based on chest CT severity score (CT-SS) (based on the degree of involvement of the lung lobes, 0%: 0 points, 1–25%: 1 point, 26–50%: 2 points, 51–75%: 3 points, and 76–100%: 4 points). The CT-SS was the sum of the scores of the five lobes (range 0–20) [19].

According to the WHO disease severity classification, patients were divided into severe and non-severe COVID-19 groups. Patients with clinical signs of pneumonia (i.e., fever, cough, and dyspnea) and SPO2≥ 90% on room air were classified as non-severe cases and patients with clinical signs of pneumonia (i.e., fever, cough, and dyspnea) and at least one of the following signs were classified as severe cases: respiratory rate> 30 breaths/minute, severe respiratory distress, SpO2 < 90% on room air, shock, or other organ failures [20].

Eosinophilia was defined as eosinophil levels≥ 2% and non-eosinophilia as eosinophil levels< 2%.

### Ethical considerations

This study has been approved by the ethics committee of Kerman University of Medical Sciences (Code: IR.KMU.AH.REC.1400.254). Written informed consent was obtained from each participant.

### Statistical analysis

Descriptive statistics (frequency, relative frequency, mean, and standard deviation), analytical statistics (chi-square test and independent t-test), and SPSS software version 20 were used to analyze the data. The significance level considered was *P*≤ 0.05.

## Results

This study included 73 COPD patients with confirmed COVID-19, with a mean age of 67.90±13.71 years. Most of the patients were male (54.8%), and the majority had hypertension (HTN) (42.5%). Twenty-seven patients had a history of smoking (27.4%), and 36 (49.3%) had a history of opium use (Table 1).

**Table 1:** Demographic characteristics of COPD patients with confirmed COVID-19 (*n*= 73)

**Variable**		**Total**		**Non-eosinophilia** **(*n* = 38)**		**Eosinophilia** **(*n* = 35)**		***P*-value**
		*N*	%	*N*	%	*N*	%	
**Gender**	**Male**	40	54.8	18	47.4	22	62.9	0.184
	**Female**	33	45.2	20	52.6	13	37.1	
**Past medical history**	**HTN**	31	42.5	16	42.1	15	42.9	0.948
	**Diabetes mellitus (DM)**	21	28.8	15	39.5	6	17.1	0.035
	**Ischemic heart disease (IHD)**	12	16.4	5	13.2	7	20	0.431
	**Chronic kidney disease (CKD)**	2	2.7	1	2.6	1	2.9	0.953
**History of smoking**		20	27.4	9	23.7	11	31.4	0.459
**History of opium use**		36	49.3	20	52.6	16	45.7	0.55

**Fig. 2:**
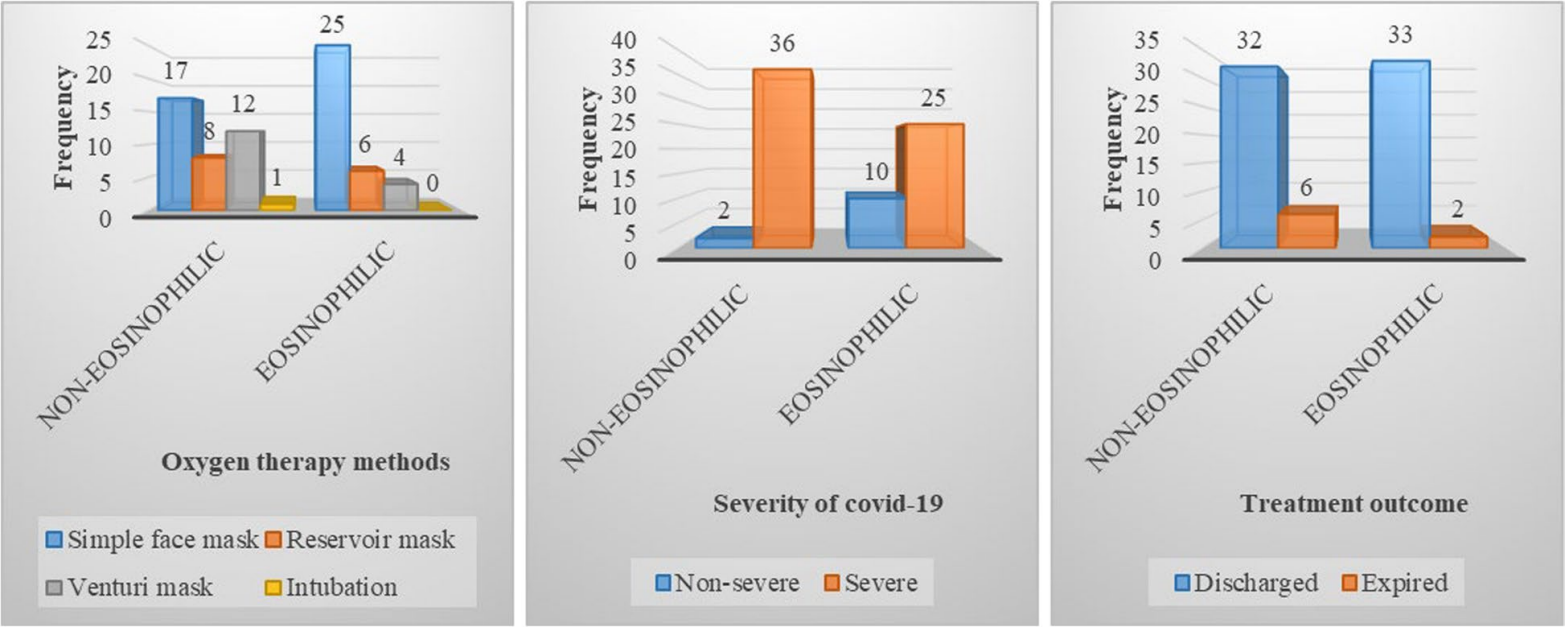
Oxygen therapy methods, the severity of COVID-19, and treatment outcomes in non-eosinophilic and eosinophilic COPD Patients (*n*= 73)

In the present study, 72 (98.6%) patients underwent noninvasive ventilation, and only one (1.4%) underwent invasive mechanical ventilation. Thirty-eight patients were in the non-eosinophilic group, and 35 were in the eosinophilic group. The mean age of non-eosinophilic and eosinophilic COPD patients was 67.55±2.37 and 68.29±2.17 years, respectively (*P=* 0.821). In both groups, most of the patients received simple facemasks. The relationship between the two groups according to oxygen therapy methods was not significant (*P=* 0.082). In the non-eosinophilic and eosinophilic groups, 94.7% and 71.4% of patients had severe COVID-19, respectively (*P=* 0.007). Mortality rate in COPD patients with COVID-19 was 11%. Non-eosinophilic COPD patients had a higher mortality rate (15.8). Most patients were discharged with good health conditions (84.2% in the non-eosinophilic and 94.3% in the eosinophilic group) (*P=* 0.169) (Fig. 2).

The average CT-SS in non-eosinophilic and eosinophilic COPD patients was 9.76±0.7 and 6.26±0.63, respectively (*P=* 0.001).

The mean of SPO2 on admission day in non-eosinophilic group was 77.5±1.81 and in eosinophilic group was 84.94±1.26. There was a significant difference in SPO_2_ on admission day (*P=* 0.001) between the eosinophilic and non-eosinophilic groups. The mean of SPO2 on discharge day in non-eosinophilic and eosinophilic groups was 93±0.68 and 95.43±0.49, respectively. There was a significant difference in SPO_2_ on discharge day (*P=* 0.006) between two groups.

**Fig. 3:**
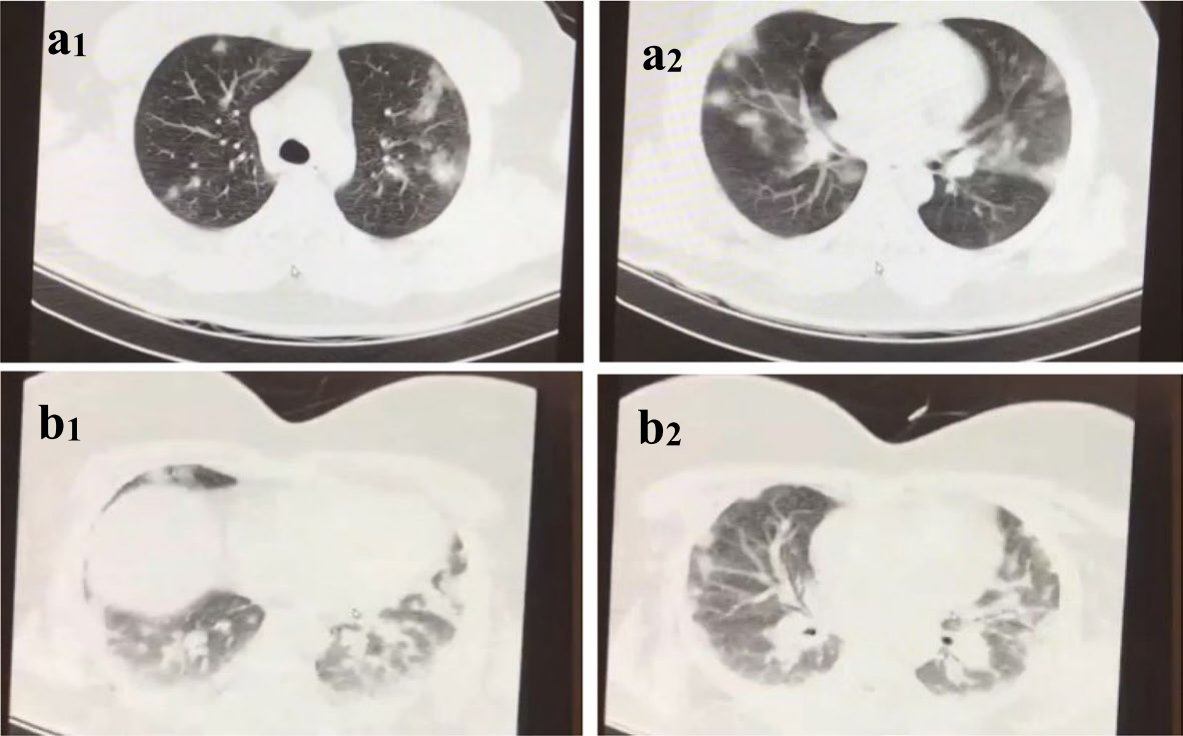
CT images of COPD patients with confirmed COVID-19. Non-severe (a_1-2_) and severe (b_1-2_)

Sixty-one cases of COPD patients had severe COVID-19 (83.6%). 94.7% of them were in the non-eosinophilic group, and 25 cases were in the eosinophilic group (71.4%). This difference was significant (0.007). This shows that COVID-19 causes more severe disease in non-eosinophilic COPD patients (Fig. 3).

In terms of laboratory tests, there were no significant differences observed in ESR, WBC, LDH, D-dimer, Hb, HCT, MCV, PLT, NEUT, NEUT/ LYMPH, and LYMPH between the eosinophilic and non-eosinophilic groups (Table 2).

**Table 2:** Comparison of CT-scan and blood parameters between non-eosinophilic and eosinophilic COPD patients (*n* = 73)

**Group**	**Non-Eosinophilic** **(*n* = 38)**		**Eosinophilic** **(*n* = 35)**		***P*-value**
**Parameters**	**Mean**	**SD**	**Mean**	**SD**	
**CT-SS**	9.76	0.7	6.26	0.63	0.001
**SPO**_**2**_** on admission day**	77.5	1.81	84.94	1.26	0.001
**SPO**_**2**_** on discharge day**^**a**^	93	0.68	95.43	0.49	0.006
**ESR (mm/h)**	41.24	6.09	42.17	5.79	0.912
**WBC (10**^**9**^**/L)**	9.58	0.90	8.92	0.83	0.595
**LDH (U/L)**	574.45	86.02	501.41	35.56	0.437
**D-dimer (mg/L)**	2.87	1.25	1.72	0.58	0.385
**Hb (g/L)**	12.38	0.33	13.67	1	0.213
**HCT (%)**	38.58	1.09	38.99	1	0.792
**MCV (fL)**	84.03	2	85.86	1.4	0.464
**PLT (10**^**9**^**/L)**	211.63	15.3	222.31	19.43	0.665
**NEUT (%)**	81.95	1.37	80.12	1.25	0.332
**NEUT/ LYMPH (%)**	12.35	1.64	12.86	2.14	0.85
**​LYMPH (%)**	9.99	1.17	10.2	0.95	0.891

^a^*n*= 65

Based on univariate logistic regression, the relationship between diabetes, oxygen therapy, severity of COVID-19, CT-SS, SPO2 on admission day and SPO2 on discharge day with eosinopenia was significant. However, based on multivariate logistic regression, none of the investigated variables was related to eosinopenia (Table 3).

**Table 3:** Logistic regression of eosinopenia for outcomes

	**Univariate**	***p*-value**	**Multivariate**	***p*-value**
	**OR (95% CI)**		**OR (95% CI)**	
**Gender**	1.88 (0.73-4.79)	0.18		
**Age**	1 (0.97-1.03)	0.81		
**HTN**	1 (0.4-2.61)	0.94		
**Diabetes mellitus (DM)**	0.04 (0.1-0.94)	0.04	0.45 (0.13-1.54)	0.2
**Ischemic heart disease (IHD)**	1.65 (0.47-5.77)	0.43		
**Chronic kidney disease (CKD)**	1.08 (0.06-18.08)	0.95		
**History of smoking**	1.47 (0.52-4.15)	0.46		
**History of opium use**	0.75 (0.3-1.9)	0.55		
**Oxygen therapy methods**	0.47 (0.26-0.86)	0.01	0.67 (0.34-1.33)	0.26
**Severity of COVID-19**	0.13 (0.02-0.64)	0.01	0.50 (0.07-3.4)	0.48
**Treatment outcomes**	0.32 (0.06-1.72)	0.18	0.29 (0.04-2)	0.21
**CT-SS**	0.79 (0.68-0.91)	0.002	0.97 (0.6-1.56)	0.92
**SPO**_**2**_** on admission day**	1.12 (1.03-1.21)	0.005	1.06 (0.85-1.31)	0.58
**SPO**_**2**_** on discharge day**	1.22 (1.04-1.42)	0.01	1.07 (0.89-1.29)	0.45
**ESR (mm/h)**	1 (0.98-1.01)	0.91		
**WBC (10**^**9**^**/L)**	0.97 (0.89-1.06)	0.59		
**LDH (U/L)**	0.99 (0.99-1)	0.45		
**D-dimer (mg/L)**	0.91 (0.75-1.11)	0.39		
**Hb (g/L)**	1.01 (0.84-1.2)	0.91		
**HCT (%)**	1.01 (0.94-1.08)	0.78		
**MCV (fL)**	1.01 (0.97-1.06)	0.46		
**PLT (10**^**9**^**/L)**	1 (0.99-1)	0.97		
**NEUT (%)**	0.97 (0.91-1.03)	0.32		
**NEUT/ LYMPH (%)**	1 (0.96-1.04)	0.84		
**​LYMPH (%)**	1 (0.93-1.07)	0.88		

## Discussion

This study was conducted on 73 COPD patients with confirmed COVID-19 to determine the relationship between peripheral blood eosinophil levels and the severity of COVID-19. In our study, most patients were discharged with good health conditions (84.2% in the non-eosinophilic and 94.3% in the eosinophilic group). The average pulmonary involvement in CT scans in COPD patients was significantly higher in the non-eosinophilic group. In addition, the average SPO_2_ on admission and discharge days in non-eosinophilic COPD patients was significantly lower than in eosinophilic COPD patients. Furthermore, the number of COPD patients with severe COVID-19 was significantly higher in the non-eosinophilic group. This shows that COVID-19 causes more severe disease in non-eosinophilic COPD patients.

We found that 83.6% of patients with COPD had severe COVID-19 (94.7% and 71.4% in non-eosinophilic and eosinophilic groups, respectively). Hansen et al. showed that patients with COPD had a significantly higher risk of developing severe COVID-19 compared to patients without COPD, with a risk difference of 4.7% [21]. The result of a meta-analysis study confirmed that the risk of developing severe COVID-19 in a patient with COPD was four times higher than in patients without COPD [22]. In a brief meta-analysis, Lippi et al. showed that COPD was associated with a significant (more than fivefold) increase in the risk of severe COVID-19 infection [23]. These findings emphasize the importance of careful control of the outbreak of COVID-19 and the urgent need for mitigation strategies in patients with COPD.

In other words, more than 94% of COPD patients with eosinophils < 2% had severe COVID-19. The study by Cosio et al. revealed that eosinopenia, along with the acute exacerbation of COPD caused by COVID-19 infection, increased the mortality and severity of COVID-19 [24]. Therefore, considering the severity of COVID-19, their results are consistent with those of our study. Hansen et al. demonstrated that low counts of blood eosinophils were associated with worse outcomes of COVID-19 in patients with COPD. As high levels of eosinophils are associated with deleterious effects in COPD, it is necessary to evaluate the mechanisms and relationships between type 2 inflammation and the outcomes of COVID-19 in prospective studies among patients with obstructive lung diseases [21]. As the results of the present study and similar studies show, patients with less than 2% eosinophils had more severe COVID-19. The reason for the severity of COVID-19 in COPD patients with non-eosinophilia may be due to type 2 inflammation. Eosinophils are a type of white blood cell that circulate in the bloodstream and can be found in various tissues. Their levels can vary in different diseases, with notable associations in conditions such as parasitic infections and allergies. They serve as immune regulatory cells that play a role in protective immunity. They have the ability to migrate to tissues, such as lung tissue, where they take up residence and carry out antiviral activities against respiratory viruses [8, 9].

In our study, most patients were discharged with good health conditions (84.2% in the non-eosinophilic group and 94.3% in the eosinophilic group), and only 11% expired. Yang et al. and Salturk et al. showed that decreased blood eosinophils were associated with increased in-hospital mortality in AECOPD patients admitted to the ICU [25, 26]. It is possible that the role of eosinophils as antibacterial and antiviral defenders in the host response is due to the release of cationic secondary granule proteins. These proteins contribute to the functions of the eosinophil in airway inflammation, tissue damage, and remodeling in inflammatory process.

The current study showed that the average CT-SS was significantly higher among non-eosinophilic COPD patients, and non-eosinophilia has a statistically significant relationship with the severity of lung parenchymal involvement in chest CT scans of COVID-19 patients. Our results are consistent with those of Zhang et al., who found that eosinopenia and lymphopenia were associated with the severity of COVID-19 [6]. In the study by Yan et al., the patients with bilateral pneumonia in their chest CT scans had significantly lower eosinophil counts than those with unilateral pneumonia [21]. This result can confirm our findings about the relationship between low eosinophil count and lung involvement in chest CT scans, i.e., it may indicate the severity of the disease. Also, In Xie et al.’s study, COVID-19 patients were evaluated in two categories: patients with eosinophil levels less than 2% (low-EOS group) and those with eosinophil levels greater than and equal to 2% (normal-EOS group) [25]. In their study, the low-EOS group had more lung involvement in chest CT scans; these results are consistent with our study results, as were other similar studies about clinical outcomes of eosinophilia in COVID-19 patients [16]. Pulmonary eosinophils can directly interact with CD8 T-cells and promote the recruitment of virus-specific CD8 T-cells into the lungs to enhance antiviral immunity. However, Eosinopenia may serve as a prognostic indicator for more severe COVID.

Although WBC, LDH, D-dimer, and NEUT were higher among non-eosinophilic COPD patients, the difference was not significant. This could be because of our small sample size.

Admission and discharge SPO2 were lower in the patients with non-eosinophilic COPD. It is worth noting that this difference was significant. However, in Valverde-Mongeet et al.’s study, no significant relationship existed between SPO2 on first admission and eosinopenia [27]. This discrepancy may have been due to the high severity of the disease in our study.

## Conclusions

Based on our research findings, COVID-19 patients with COPD who exhibit lower peripheral blood eosinophil levels tend to experience more unfavorable outcomes. Our analysis indicated that non-eosinophilia in hospitalized patients may be linked to disease severity and lung parenchymal involvement, increasing the likelihood of requiring noninvasive and invasive oxygen therapy and raising mortality risks. In addition, non-eosinophilia in COPD patients with COVID-19 causes more frequent hospitalizations.

## Limitations

The diagnosis of COPD is based on the PFT test. Because we examined the patients with COVID-19, it was not possible to perform the PFT test. During the acute period of COVID-19 pneumonia, it was not possible to perform spirometry due to the contagiousness of the disease and the limited cooperation of the patient, who had shortness of breath and tachypnea. Therefore, the diagnosis of COPD was made based on the past medical history or clinical examination. Another limitation of our study may be due to the small sample size, which is why we did not reach statistically significant results in some variables. Also, it is one of the limitations of study that the vaccination history of the patients in the study was not evaluated.

## Data Availability

All data generated or analyzed during this study are included in this published article.
